# Comparison of oral versus intravenous methadone on postoperative pain and opioid use after adult spinal deformity surgery: A retrospective, non-inferiority analysis

**DOI:** 10.1371/journal.pone.0288988

**Published:** 2023-07-21

**Authors:** Kamilla Esfahani, William Tennant, Siny Tsang, Bhiken I. Naik, Lauren K. Dunn

**Affiliations:** 1 Department of Anesthesiology, University of Virginia, Charlottesville, Virginia, United States of America; 2 Department of Neurological Surgery, University of Virginia, Charlottesville, Virginia, United States of America; Beth Israel Deaconess Medical Center / Harvard Medical School, UNITED STATES

## Abstract

**Objective:**

To compare efficacy of oral versus intravenous (IV) methadone on postoperative pain and opioid requirements after spine surgery.

**Methods:**

This was a retrospective, single-academic center cohort study evaluating 1010 patients who underwent >3 level spine surgery from January 2017 to May 2020 and received a one-time dose of oral or intravenous methadone prior to surgery. The primary outcome measured was postoperative opioid use in oral morphine equivalents (ME) and verbal response scale (VRS) pain scores up to postoperative day (POD) three. Secondary outcomes were time to first bowel movement and adverse effects (reintubation, myocardial infarction, and QTc prolongation) up to POD 3.

**Results:**

A total of 687 patients received oral and 317 received IV methadone, six patients were excluded. The IV group received a significantly greater methadone morphine equivalent (ME) dose preoperatively (112.4 ± 83.0 mg ME versus 59.3 ± 60.9 mg ME, p < 0.001) and greater total (methadone and non-methadone) opioid dose (119.1 ± 81.4 mg ME versus 63.9 ± 62.5 mg ME, p < 0.001), intraoperatively. Although pain scores for the oral group were non-inferior to the IV group for all postoperative days (POD), non-inferiority for postoperative opioid requirements was demonstrated only on POD 3. Based on the joint hypothesis for the co-primary outcomes, oral methadone was non-inferior to IV methadone on POD 3 only. No differences in secondary outcomes, including QTc prolongation and arrhythmias, were noted between the groups.

**Conclusions:**

Oral methadone is a feasible alternative to IV methadone for patients undergoing spine surgery regarding both pain scores and postoperative opioid consumption.

## Introduction

Finding the optimal analgesic regimen for complex spine surgery remains challenging, especially regarding opioids and opioid-sparing approaches. Intraoperative exposure to opioids like fentanyl, sufentanil, or remifentanil have been shown to result in higher pain scores and opioid consumption in the postoperative period [1–3]. Methadone is a long-acting opioid agonist and N-methyl-D-aspartate receptor antagonist with a long elimination half-life (range of 13–50 hours, mean of 24–36 hours) and has shown benefit for postoperative analgesia in spine surgery patients [4, 5]. In a prospective, randomized trial of 29 patients, a single dose of intravenous (IV) methadone reduced postoperative pain scores and opioid requirements at 48 hours after surgery compared to IV sufentanil [6]. More recent studies demonstrated that a single dose of IV methadone can provide improved postoperative pain control and reduced opioid consumption through postoperative day two and up to one year after surgery [7, 8]. Despite these benefits, use of IV methadone has been limited due to concerns that its long duration of action may lead to significant side effects, such as sedation, respiratory depression, ileus, nausea, and vomiting.

Oral pain management prior to surgery can effectively reduce postoperative pain [9]. During a nationwide IV opioid shortage, Salajegheh et al compared the effect of oral opioids administered compared to intravenous opioids on patient outcomes at our institution [10]. The results showed no difference in pain scores between the two groups, despite oral opioids being used more commonly than IV. Moreover, patients in the IV group received greater total opioids, overall. These findings and the development of enhanced recovery protocols has led to increasing use of oral analgesic medications for perioperative analgesia, as well as an increasing focus on safe and cost-effective patient care. The cost differential between an equivalent dose of IV methadone aliquoted into a prefilled syringe from a 20 mL multi-dose vial by our operating room pharmacy compared to oral methadone tablet is 1000-fold [11]. IV methadone is routinely used at our institution for perioperative analgesia for major spine surgery [6]; however, differences in analgesic effects and postoperative outcomes between oral and IV methadone have not been studied.

Here, we compared the impact of a single preoperative dose of oral versus IV methadone for patients undergoing spine surgery, using a joint hypothesis testing approach for reduction in postoperative pain and opioid consumption. We hypothesized that oral methadone would be non-inferior to IV methadone for both postoperative pain and opioid consumption.

## Methods

### Study design and approval

This study was approved by the University of Virginia Institutional Review Board for Health Science Research (UVA IRB HSR #22498). The requirement for written consent was waived. This study adheres to the Strengthening the Reporting of Observational studies in Epidemiology (STROBE) guidelines for reporting of observational data.

### Study sample

The study sample was a convenience sample. Adult patients (age ≥ to 18 years and < 90 years of age) who underwent elective, > 3 level posterior spinal fusion between July 3, 2017 and July 23, 2020, and received either oral or IV methadone immediately prior to surgery were identified and the electronic medical records reviewed. We identified a total of 1010 patients; 317 (31.4%) patients received IV methadone and 687 (68.0%) were given oral methadone. Six received both IV and oral methadone and were excluded from subsequent analyses.

### Exposure variable

Each patient received either a single dose (mg) of oral methadone preoperatively or a single dose (mg) of IV methadone in the operating room prior to onset of surgery. Given the retrospective nature of the study, standardization of prescribed opioids was not feasible. The methadone dose administered preoperatively was recorded and subsequently converted to morphine equivalent (mg ME) dose using the CDC morphine milligram equivalents table and a conversion ratio of 1:2 for IV to oral methadone [12, 13]. The morphine equivalent conversion is not fixed, with larger doses having an additional correction factor, as illustrated in the CDC table. For example, a 20 mg oral methadone dose was multiplied by 4 morphine equivalents to yield a total of 80 mg ME. For a 25 mg oral methadone dose, the first 20 mg were multiplied by 4. The remaining 5 mg were multiplied by 8, for a total of 120 mg ME. Methadone dose (in mg ME) were combined with non-methadone opioids (in mg ME) administered during surgery to yield a total opioid dose (in mg ME).

Our institution had no specific protocols regarding methadone dosing. Dosing and formulation of the drug were at the discretion of the attending anesthesiologist. Oral methadone was administered in the preoperative area, at the time of patient evaluation (approximately 30 minutes prior to initiating intraoperative care).

### Primary and secondary outcomes

Co-primary outcomes included postoperative opioid consumption, reported as a postoperative morphine equivalents (ME) and an 11-point patient-reported verbal rating scale (VRS) pain scores (0–10), measured on postoperative days 1–3. Secondary outcomes included time to first bowel movement (days), reintubation rates, postoperative myocardial infarction, or new onset arrhythmia and new QTc prolongation.

### Study variables

Data collected included age, sex, body mass index (BMI), and American Society of Anesthesiologists (ASA) physical status classification, preoperative opioid use, preoperative vital signs, preoperative and intraoperative non-opioid medication use. Patient’s age, sex, BMI, ASA class, preoperative opioid use, and intraoperative medications were included as covariates in the models. An a priori sensitivity analyses was planned to evaluate patients left intubated postoperatively using the critical care postoperative observation tool (CPOT) score.

### Intraoperative and postoperative management

Standard perioperative management included induction of anesthesia with IV lidocaine (1–1.5 mg/kg), propofol (1–2 mg/kg), and rocuronium (0.5–1 mg/kg). Anesthesia was maintained with IV infusions of propofol (50–150 mcg/kg/min), lidocaine (40 mcg/kg/min), and ketamine (0.3–0.5 mg/kg/h) with or without up to one half minimum alveolar concentration of volatile anesthetic. Patients received IV hydromorphone towards the end of the procedure at the discretion of the anesthesiologist. Postoperative analgesia included hydromorphone patient-controlled analgesia and oral analgesics. Patients were transferred to the intensive care unit (ICU) for postoperative monitoring.

### Statistical analysis

Categorical data between the oral versus IV methadone group were analyzed using the Chi-square test or Fisher’s exact test. Count data was compared using the generalized linear regression model with the Poisson link. Continuous data (expressed as mean ± standard deviation (SD) or median [25^th^-75^th^ quartile]) were analyzed using the Wilcoxon rank sum test or linear regression models (with group: oral vs. IV as independent variable), where appropriate. We used linear regression models, controlling for preoperative methadone dose and covariates (age, sex, BMI, ASA class, preoperative opioid use, and intraoperative medications) to examine the extent to which postoperative pain scores and postoperative opioid consumption were different between those who received IV or oral methadone. Three sets of linear regression models were performed for average pain score, maximum pain score, and postoperative opioid consumption as the dependent variable, separately for POD0 to POD3. An additional set of subgroup linear regression models was performed using postoperative CPOT scores as the dependent variable, separately for POD0 to POD3. In all models, group (oral vs. IV methadone) was included as an independent variable, with IV methadone group used as the reference group. All models controlled for preoperative methadone dose and covariates listed above. Traditional assumptions of linear regression models, including linearity, homoscedasticity, and independence and normality of residuals are met. Pain scores were square root transformed, whereas opioid consumption was log-transformed due to skewness of the data. Estimated results were back-transformed for ease of interpretation.

The non-inferiority margin for pain scores was defined a priori as a Δ = 1 point, and the equivalence margin for pain scores was defined as a Δ = 1 point in both directions. The non-inferiority margin for morphine equivalent was defined as not more than 20% greater than IV, and the equivalence margin was defined as no more than 20% greater or less than the IV methadone group.

For the two-step joint hypothesis gatekeeping procedure for both pain scores and opioid consumption, we considered oral methadone to be non-inferior to IV methadone if: 1) Oral methadone was non-inferior on pain score (maximum or average) and non-inferior on morphine equivalent consumption; or 2) Oral methadone was non-inferior on pain scores (maximum or average) and equivalent on morphine equivalent consumption; or 3) Oral methadone was equivalent on pain scores (maximum or average) and non-inferior on morphine equivalent consumption.

Finally, a subgroup analysis of differences in CPOT scores for patients not extubated after surgery, between the oral and IV methadone group, was performed using a linear regression model controlling for the same covariates as previously described. CPOT was square root transformed in the analyses, and back-transformed results were presented for interpretation.

As this was a convenience sample, no a priori power analysis was performed. A p value < 0.05 was considered statistically significant for all analyses. No adjustment for multiple comparisons were used. All statistical analyses were conducted in R (version 4.0.3).

## Results and discussion

Preoperative and intraoperative patient characteristics are shown in Table 1. There were significant differences in ASA physical status, preoperative opioid use (IV methadone: 83% vs. oral methadone: 94%, p < 0.001) and gabapentin use (IV methadone: 2.5% vs. oral methadone: 14.5%, p < 0.001). The oral methadone group received lower total doses of ketamine, lidocaine, propofol and dexmedetomidine intraoperatively compared to the IV methadone group. There was a significant difference in the median methadone dose between groups [oral methadone 40 mg ME (IQR 40–80) vs. IV methadone 80 mg (IQR 80–80), p < 0.001]. Total mean opioid morphine equivalents, which included both methadone and non-methadone opioids administered intraoperatively, were significantly higher in the IV methadone group (IV: 119.1 ± 81.4 mg vs. Oral: 63.9 ± 62.5 mg, p < 0.001).

**Table 1:** Preoperative characteristics and opioid usage.

**Preoperative Characteristics**	**IV (N = 317)**	**Oral (N = 687)**	**P value**
Age^1^ (years)	65.1 ± 12.35	64.8 ±11.4	0.757
Sex^3^			0.715
Female	161 (50.8%)	359 (52.3%)	
Male	156 (49.2%)	328 (47.7%)	
ASA class^4^			< .001
1	2 (0.1%)	6 (0.9%)	
2	96 (30.3%)	289 (42.1%)	
3	206 (65.0%)	385(56.0%)	
4	12 (3.8%)	7 (0.01%)	
Preoperative Opioid Use^3^			<0.001
No	53 (16.7%)	40 (5.8%)	
Yes	264 (83.3%)	647 (94.2%)	
Body Mass Index^1^, kg/m^2^	31.2 ±6.5	31.2 ±6.7	0.973
Systolic Blood Pressure^1^ (mmHg)	139 ± 203	139 ± 21	0.665
Diastolic Blood Pressure^1^ (mmHg)	73 ± 11	74 ± 10	0.231
Oxygen Saturation^1^ (%)	97 ± 2	97 ± 2	0.004
Heart Rate^1^ (bpm)	76 ± 13	73 ± 12	<0.001
Temperature^1^ (degree Fahrenheit)	98.1 ± 0.5	98.0 ± 0.4	0.447
Respiratory Rates^1^	17 ± 21	17 ± 2	0.830
Preoperative medications^3^			
Acetaminophen	93 (29.3%)	202 (29.4%)	1
Gabapentin	8 (2.5%)	100 (14.6%)	<0.001
Benzodiazepines	181 (57.1%)	348 (50.7%)	1
**Intraoperative Characteristics**			
Ketamine^2^ (mg)	80.2 ±271.8	58.6 ± 53.5	<0.001
Lidocaine^2^ (mg)	985.2 ± 747.7	895.6 ± 583.5	<0.001
Propofol^2^ (mg)	4875.3 ± 46105.7	1680.7 ± 1492.4	<0.001
Dexmedetomidine^2^ (mcg)	16.9 ± 53.7	16.3 ± 60.7	<0.001
**Opioids used**	**IV (N = 317)**	**Oral (N = 687)**	**P value**
**Dose of methadone**^2^ **(mg ME)**			
Mean ± SD	112.4 ± 83.0	59.3 ± 60.9	<0.001
Median (IQR)	80 (80–80)	40 (40–80)	<0.001
**Dose of non-methadone opioids**^2^ **(mg ME)**			
Mean ± SD	6.7 ± 12.2	4.57 ± 10.5	<0.001
Median (IQR)	0 (0–10.50)	0 (0)	<0.001
**Total Opioids used**^2^ **(mg ME)**			
Mean ± SD	119.1 ± 81.4	63.9 ± 62.5	<0.001
Median (IQR)	80 (80–108.50)	41 (40–80)	<0.001

^1^ Linear regression models were used for group comparisons.

^2^ Mann-Whitney-Wilcox tests were used for group comparisons.

^3^ Chi-square tests were used for group comparisons.

^4^ Fisher’s exact tests were used for group comparisons.

Preoperative demographic characteristics, vital signs, and preoperative and intraoperative medications for oral versus intravenous methadone groups. [IV–intravenous; ASA—American Society of Anesthesiologists physical status classification system, bpm—beats per minute; preoperative medications—number of patients who received oral preoperative medications; ME morphine equivalents, Total opioids–referring to methadone and non-methadone opioid morphine equivalents]. Results reported as mean ± standard deviation, median and interquartile ranges, or number (proportion) of patients.

### Postoperative pain and opioid consumption

**Fig 1:**
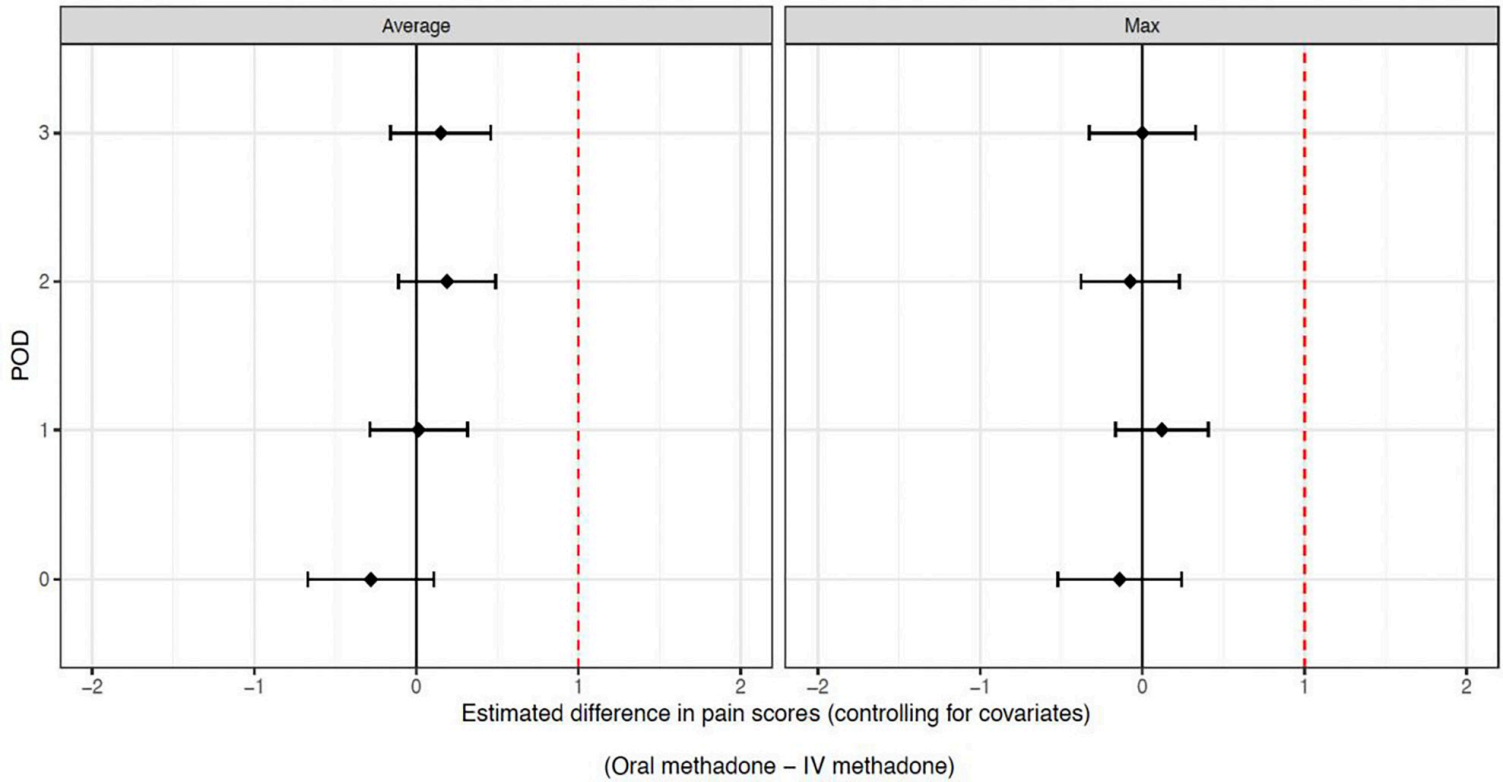
Differences in average and maximum postoperative pain scores controlling for methadone dose and covariates. The black diamond reflects the point estimate, and the whiskers represent the 95% confidence intervals for mean difference in pain scores. The dotted vertical line denotes the 1-point non-inferiority margin with non-inferiority demonstrated if the 95% confidence interval whiskers do not cross the red vertical line.

Table 2 and Fig 1 show the results of linear regression models examining the extent to which postoperative pain scores differed between patients who received IV versus oral methadone, controlling for methadone dose and covariates. Oral methadone was non-inferior and equivalent to IV methadone on both average and maximum pain scores on POD 0 to POD 3.

**Table 2:** Postoperative pain scores and opioid consumption.

POD	Average Pain Score^1^			Maximum Pain Score^1^			Postoperative Opioid Consumption (ME)^2^		
	Est	95% CI	P value	Est	95% CI	P value	Est	95% CI	P value
0	-1.84	-6.35, 2.67	0.142	-1.21	-6.20, 3.79	0.569	1.36	1.01, 1.83	0.045
1	0.54	-2.81, 3.89	0.863	1.49	-2.58, 5.55	0.286	1.35	1.10, 1.66	0.004
2	1.55	-1.67, 4.77	0.143	-1.02	-5.14, 3.10	0.621	1.11	0.87, 1.41	0.405
3	1.10	-2.11, 4.32	0.46	-0.56	-4.79, 3.68	0.886	0.84	0.62, 1.14	0.252

^1^Pain scores were square root transformed in the analyses, results presented here are back-transformed for ease of interpretation.

^2^ Postoperative opioid consumption was log-transformed in the analyses, results presented here are back-transformed for interpretation.

Estimates from linear regression models estimating the differences in average and maximum pain score and opioid consumption between intravenous and oral methadone groups, controlling for methadone dose and covariates. A positive estimated difference indicates more pain or more opioid consumption in the oral methadone group compared to IV methadone group. [POD–postoperative day number; ME–morphine equivalents; Est–estimated difference (back-transformed); CI–confidence interval]

**Fig 2:**
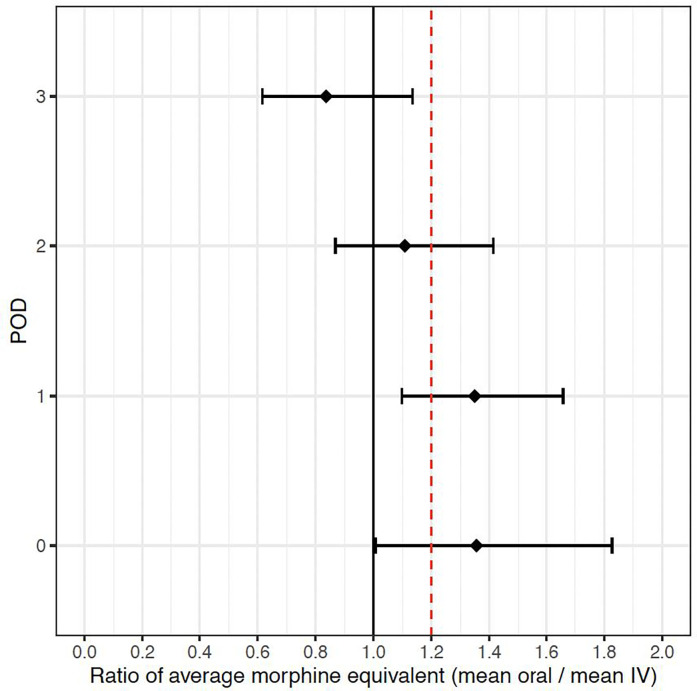
Ratio of postoperative mean oral morphine equivalents divided by intravenous morphine equivalents controlling for covariates and methadone dose. The black diamond reflects the point estimate ratio and the whiskers represent the estimated 95% confidence intervals. The dotted vertical line denotes the 1.2 ratio non-inferiority margin with non-inferiority demonstrated if the 95% confidence interval does not cross the red vertical line.

Regarding opioid consumption, oral methadone only demonstrated non-inferiority on POD 3. Therefore, based on our a priori two-step joint hypothesis approach, non-inferiority of oral methadone compared to IV methadone was only achieved on POD 3 for opioid consumption (Table 2 and Fig 2).

We also examined the extent to which CPOT scores for patients who remained intubated postoperatively were different between IV and oral methadone groups (Table 3) in a sub-group analysis. After controlling for the above covariates, there were no observed differences in CPOT scores between the IV and oral groups.

**Table 3:** Postoperative CPOT scores.

POD	CPOT	Est	95% CI	P value
0	Average	-1.83	-6.17, 2.52	0.134
	Max	-1.72	-6.87, 3.43	0.26
1	Average	-1.08	-3.65, 1.48	0.371
	Max	-2.07	-6.58, 2.44	0.063
2	Average	-0.28	-3.49, 2.94	0.963
	Max	0.65	-5.05, 6.35	0.884
3	Average	-0.56	-3.92, 2.80	0.855
	Max	-1.12	-6.82, 4.58	0.667

Unstandardized estimates from linear regression models estimating the differences in CPOT score between IV and oral methadone groups controlling for patient age, sex, BMI, ASA class, preoperative opioid use, and intraoperative medication administration, as well as methadone dose. CPOT scores were square root transformed in the analyses, results presented here are back-transformed for ease of interpretation. [POD—postoperative day number; CPOT—critical care postoperative observation tool score; Est—estimate; CI—confidence interval]

### Secondary outcomes

Descriptive statistics for secondary outcomes including time to first bowel movement, reintubation, postoperative myocardial infarction (MI), postoperative arrhythmia, and QTC prolongation are shown in Table 4. Results showed that the oral methadone group had ~10% lower incident rate for time to first bowel movement than the IV methadone group. No other significant differences were observed between the groups.

**Table 4:** Comparison of secondary outcomes between IV and oral methadone groups.

Secondary Outcomes	IV	Oral	P value
Time to first BM	3 [2, 4] Range = 1–11	3 [2, 4] Range = 0–10	0.010
Reintubation	3 (0.95%)	5 (0.73%)	0.71
Postoperative MI	2 (0.63%)	3 (0.44%)	0.65
Postoperative Arrhythmia	20 (6.3%)	30 (4.4%)	0.24
QTC prolongation	68 (21.5%)	150 (21.8%)	0.10

Described by the number or proportion of patients experiencing each outcome. Generalized linear regression with Poisson link was used for Time to first BM variable, and Chi-square/Fisher’s test were used for the remaining variables. Time to first BM is reported as median [IQR] and range. The rest of the variables are reported as count and percent of total IV or total Oral group. [BM- bowel movement; SD–standard deviation; MI—myocardial infarction; QTC—corrected QT interval on electrocardiogram].

## Discussion

Based on our a priori joint hypothesis approach of both pain scores and opioid consumption, our study demonstrates that oral methadone was non-inferior to IV methadone on POD 3. The literature has extensively evaluated the perioperative analgesic benefits of IV methadone in various surgeries, such as cardiac and spine, but is limited in describing the impact of oral methadone compared to IV. One randomized controlled-trial studied the effects of oral methadone compared to placebo on postoperative analgesia outcomes for sternotomy pain in patients undergoing coronary artery bypass graft surgery [14]. Twenty-one patients were randomized to receive standard care with one dose of preoperative oral methadone (n = 9) or standard care with placebo (n = 12), with an oral liquid methadone to IV conversion ratio of 1:1. They excluded patients who had prolonged preoperative QTc on EKG, pulmonary disease requiring home oxygen supplementation, had a recent history of opioid use disorder, or were opioid tolerant patients (oral morphine equivalents of 60 mg daily for one week or longer). A single dose of preoperative methadone significantly reduced postoperative opioid consumption, but showed equivalent pain scores and similar side-effect profiles (up to 72 hours).

Our study included patients with opioid use history and used linear regression models to account for variability between IV and oral methadone group. This retrospective, observational study found no difference in pain scores between the two groups, but a significant reduction in opioid requirements on POD 3 for the oral methadone group (Table 2). We also observed no difference in patients whose surgeries were complicated by prolonged intubation (Table 3).

To account for this significant finding, we can consider multiple factors, including pharmacological differences in route of medication. Pharmacokinetic studies have demonstrated that a single dose of 20 mg IV methadone provides long duration of analgesia with minimal risk of respiratory depression [5]. The mean doses of methadone administered fall within this safe therapeutic window, and the incidence of adverse events was low. Plasma levels of 58 ng/mL after a single dose of methadone have been shown to provide effective pain relief after many surgery types [15]. More recent studies demonstrated that a single dose of 0.2 mg/kg IV administered preoperatively may provide opioid sparing effects lasting up to a year out of surgery [6, 8]. In the outpatient surgical setting, Komen et al. found that a one-time intraoperative IV methadone bolus (0.15 mg/kg ideal body weight) decreased perioperative opioid consumption and pain scores and resulted in a similar side-effect profile to patients who received conventional opioids [16]. These results support the one-time dosing regimen used in our study.

The findings of our study might be related to different pharmacokinetic profiles for oral vs. IV methadone. Equianalgesic dosing for oral and IV methadone is highly variable due to differences in pharmacokinetics and pharmacogenomics. Compared to oral methadone, the intravenous formulation is much more potent. Methadone binds to alpha-1-glycoprotein and is metabolized by cytochrome P450 enzymes. Liver disease may alter alpha-1-glycoprotein levels, which in turn influences methadone’s unbound serum concentrations. Variability in the cytochrome P450 enzymes impacts methadone plasma concentrations as well. Genetic polymorphisms in CYP2B6, the primary enzyme involved in methadone metabolism and clearance, can result in slow to rapid metabolizers [17–20]. A significant proportion (approximately 20%) of oral methadone undergoes first-pass metabolism by intestinal activity. Oral bioavailability can therefore vary greatly between individuals [21, 22]. Absorption kinetics for oral methadone are limited. Future studies comparing oral vs. IV methadone would benefit from simultaneous analysis of plasma methadone concentrations.

While this observational study’s primary goal was not focused on cost-effectiveness, the results could influence future directions in research as more hospitals focus on cost-saving and enhanced surgical outcomes. Non-inferiority of the oral formulation could influence preoperative multimodal analgesia choices, especially in Enhanced Recovery After Surgery (ERAS) programs. ERAS protocols are widely accepted as key tools to reduce morbidity and mortality in patients across multiple surgical specialties, including spine surgery. Many of these protocols have successfully utilized multimodal oral medications to reduce surgical stress and limit postoperative opioid consumption [23–25]. The return on investment in an ERAS program in a hospital system results because of decreased length of stays (LOS) and improved cost savings, such as by standardizing pain management strategies for patients [23, 26].

Salajegheh et al. found that during a national opioid-shortage, post-operative patient pain scores were similar when comparing IV to PO perioperative analgesia, while using less opioids overall in the PO group [10]. Similarly in our study, the oral group received significantly lower doses of methadone and total opioids with non-inferior results. The generalizability and safety profile of methadone needs to be further elucidated in well-powered studies. But the use of oral opioids compared to intravenous could lead to less opioid usage overall, and therefore limit side-effect profiles.

ERAS protocols vary between institutions with medication and facilities costs not standardized across health-systems. A retrospective, single-center cost-saving analysis of ERAS vs standard care in spine surgery performed by Naik et al, found that standard care was more cost-effective compared to ERAS due to a reduced LOS in the standard treatment group [26]. The findings of this study highlight the importance of evaluating all components of an ERAS program, to determine the incremental cost effectiveness of individual components. While oral medications are generally cheaper than IV formulations, careful analysis of the benefit of oral formulations is needed for methadone.

Our study has several limitations. Due to the retrospective study design, perioperative analgesia was not standardized and was at the discretion of the anesthesiologist. We could also not account for metabolism differences in patients with liver disease, which could contribute to the variable effects of oral methadone. Our study cohort included a small sample size of inpatients who were monitored in the intensive care unit postoperatively. Secondary outcomes were evaluated only up to POD 3, when acute postoperative pain usually decreases. Long-term impact of oral compared to IV methadone on pain scores and opioid utilization is unknown. More studies are needed to compare the long-term outcomes of oral methadone compared to IV. While we did not see significant differences in secondary outcomes such as respiratory depression (Table 4), due to methadone’s pharmacokinetic and pharmacogenomic variability and risk for delayed respiratory depression, appropriate monitoring is necessary. The amount of non-opioid analgesics was greater in the oral group. While data on the effectiveness of gabapentin on postoperative pain management vary [27, 28], increased gabapentin use in the oral group may have confounded our results. The patients in our study were inpatients that were closely monitored in the intensive-care unit. The data on methadone’s safety profile in the perioperative setting does not look at oral formulation, and so further studies are needed to address oral methadone safety in the perioperative setting.

## Conclusion

Our study also demonstrates that oral methadone is both non-inferior and equivalent to IV methadone for both average and maximum pain scores on all POD days and was non-inferior for opioid consumption on POD 3. This analysis suggests that oral methadone is a suitable alternative to IV methadone for patients undergoing multi-level spine surgery.
